# A comment-driven evidence appraisal approach to promoting research findings into practice when only uncertain evidence is available

**DOI:** 10.1186/s12961-023-00969-9

**Published:** 2023-03-27

**Authors:** Shuang Wang, Halil Kilicoglu, Jian Du

**Affiliations:** 1 National Institute of Health Data Science, Peking University, Beijing, China; 2 School of Information Sciences, University of Illinois at Urbana-Champaign, Champaign, USA

**Keywords:** Evidence–comment network, Scientific commentary, Evidence appraisal, Sentiment analysis, Evidence-based policy-making

## Abstract

**Background:**

Comments in PubMed are usually short papers for supporting or refuting claims, or discussing methods and findings in original articles. This study aims to explore whether they can be used as a quick and reliable evidence appraisal instrument for promoting research findings into practice, especially in emergency situations such as COVID-19 in which only missing, incomplete or uncertain evidence is available.

**Methods:**

Evidence–comment networks (ECNs) were constructed by linking COVID-19-related articles to the commentaries (letters, editorials or brief correspondence) they received. PubTator Central was used to extract entities with a high volume of comments from the titles and abstracts of the articles. Among them, six drugs were selected, and their evidence assertions were analysed by exploring the structural information in the ECNs as well as the sentiment of the comments (positive, negative, neutral). Recommendations in WHO guidelines were used as the gold standard control to validate the consistency, coverage and efficiency of comments in reshaping clinical knowledge claims.

**Results:**

The overall positive/negative sentiments of comments were aligned with recommendations for/against the corresponding treatments in the WHO guidelines. Comment topics covered all significant points of evidence appraisal and beyond. Furthermore, comments may indicate the uncertainty regarding drug use for clinical practice. Half of the critical comments emerged 4.25 months earlier on average than the guideline release.

**Conclusions:**

Comments have the potential as a support tool for rapid evidence appraisal as they have a selection effect by appraising the benefits, limitations and other clinical practice issues of concern in existing evidence. We suggest as a future direction an appraisal framework based on the comment topics and sentiment orientations to leverage the potential of scientific commentaries supporting evidence appraisal and decision-making.

**Supplementary Information:**

The online version contains supplementary material available at 10.1186/s12961-023-00969-9.

## Contributions to the literature

- We revealed that the sentiment orientation (positive/negative) of comments is largely aligned with the recommendations for specific interventions for COVID-19 in WHO guidelines.
- Comment topics were found to cover all significant points of evidence appraisal and beyond.
- Half of the critical comments emerged 4.25 months on average earlier than the guideline release.
- We show the consistency, coverage and time-efficiency of comments as an evidence appraisal tool.
- We suggest that a rigorous comment-based evidence appraisal from the perspective of comment sentiment and comment points can leverage the potential of scientific commentaries in evidence appraisal and decision-making.

## Background

Implementation science seeks to promote the uptake of research and other evidence-based findings into practice [1]. Evidence-based policy-making in healthcare relies primarily on clinical practice guidelines and systematic reviews, which synthesize high-quality primary evidence. Decision-making is always complex and involves uncertainty [2], especially when the scientific evidence is incomplete [3, 4]. For example, in the early days of the COVID-19 global pandemic, practitioners needed to make rapid therapeutic decisions from incomplete, uncertain and even conflicting scientific evidence [5, 6]. To address this problem, “living evidence” was proposed as a novel evidence synthesis process to overcome the out-of-date weakness in developing and implementing systematic reviews and guidelines in practice. Compared to the traditional approach of systematic reviews and meta-analysis by identifying and combining data across studies, the “living evidence” approach can better serve the needs of decision-makers by developing both rigorous and updated evidence summaries [7]. This approach is more appropriate “when research evidence is emerging rapidly, current evidence is uncertain and new research might change policy or practice” [8]. A living guideline allows clinicians to make individual, up-to-date recommendations by incorporating new published living evidence, which is labour-intensive and in need of an automated evidence monitoring process [9]. How to conduct rapid evidence appraisal to ensure its rigour is still a challenge for evidence synthesizers and decision-makers.

Evidence appraisal, the critical evaluation of published studies, plays an important role in differentiating rigorous science from weak science [10, 11]. To achieve consistently rigorous and updated evidence summaries, it is necessary to have a rapid and clear understanding of the characteristics (e.g. strengths, flaws and applicability) of current evidence. This echoes the idea of “meta-knowledge”, which is described as “the knowledge of knowledge”, by critically scrutinizing what is known in order to understand the current level of scientific knowledge [12]. Meta-knowledge analysis enables a better understanding of existing knowledge by, for example, re-examining and re-weighting former certainties of knowledge claims. The formally published comments on prior studies provide rich evaluative information by expressing supportive or contradictory opinions on the current evidence, but this information remains underutilized [13, 14].

Published research commentaries are formal and short communications such as letters to the editor and editorials that reflect commenters’ viewpoints by neutrally commenting on, supporting or challenging research publications [15–17]. Such commentary plays a critical post-publication role in inspecting and shaping clinical knowledge [13–15, 18, 19]. However, to our knowledge, there are few studies on how exactly research commentaries are used in clinical evidence appraisal and to what extent they shape clinical evidence. Most recently, the coevolution of evidence and practice on COVID-19 has been demonstrated by linking policy documents and the cited scientific publications [20, 21]. Nevertheless, these studies failed to reveal the detailed coevolution between scientific evidence and policy recommendations, such as the selection mechanisms for the included evidence. Informatics approaches have the potential to assist the evidence appraisal process and improve the rigour and value of clinical evidence [11]. Using the publication–comment linkages available in the PubMed database [22], this study aims to explore whether comments can support quick and reliable evidence appraisal, especially in emergency situations like COVID-19 in which only missing, incomplete or uncertain evidence is available.

We pose the following research questions:

1. Are the sentiment orientations in commentaries consistent with the strength of recommendations in clinical practice guidelines?
2. Are the topics in commentaries aligned with the core concerns of evidence appraisal in developing clinical guidelines (e.g. methodological issues of evidence, clinical adaptability and other ethical or economic issues)?
3. Do critical comments provide a faster approach to shaping the evidence than the released guideline recommendations?

## Methods

**Fig. 1:**
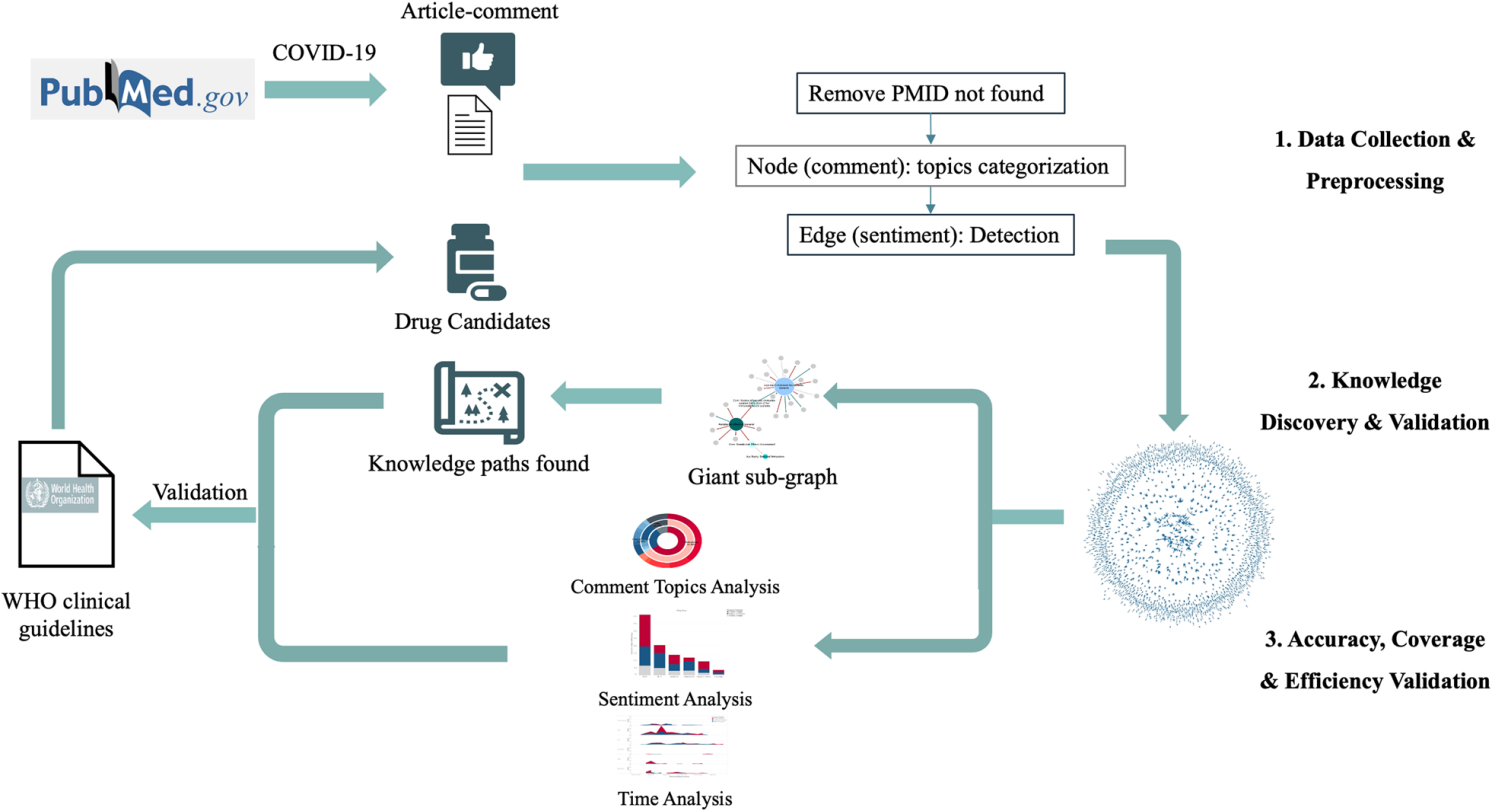
Workflow diagram

A workflow diagram of our approach is shown in Fig. 1.

### Data collection

To identify COVID-19-related publications and comments, PubMed was queried in two steps on 21 July, 2021:

- Keyword “(Covid-19[MeSH] OR Covid-19[Title/Abstract]) and hascommentin”: identifying publications (evidence) that have COVID-19 in titles or abstracts and include at least one comment (*N* = 5379)
- Keyword “(Covid-19[MeSH] OR Covid-19[Title/Abstract]) and hascommenton”: identifying published comments that have COVID-19 in titles or abstracts and comment on at least one publication (*N* = 5863)

After extracting COVID-19 evidence–comment pairs, we explored the concepts that were highly commented on. Details are provided in the supplemental material (Additional file 1). To investigate the role that comments played in evidence appraisal, we referred to WHO’s *Therapeutics and COVID-19: living guideline* [23] (five versions) and found five matched drugs (hydroxychloroquine [HCQ], IL-6 receptor blockers, remdesivir, lopinavir/ritonavir [LPV/r] and corticosteroids). As ivermectin was included in the WHO guidelines, we also added it into our study list, for a total of six drugs (corticosteroids, remdesivir, HCQ, LPV/r, ivermectin and IL-6 receptor blockers). WHO recommended using corticosteroids and IL-6 receptor blockers in severe COVID-19 and against using HCQ, remdesivir, LPV/r and ivermectin. Information on the adherence to reporting guidelines of the present research is provided in Additional file 2.

### Data preprocessing

We extracted 448 evidence–comment pairs whose titles included any of the six drug names. Two reviewers (SW and QYG) first read all 56 full texts of comments regarding corticosteroids and labelled the topics of interest and sentiment orientations for each comment separately. After the initial annotation of corticosteroids was completed, a group meeting was held with a third reviewer (JD), who reconciled disagreements. Finally, an annotation guideline was completed, and a reviewer (SW) labelled the remaining 320 comments.

Comment topics were categorized based on Kastner et al.’s categorization frame of “letter to the editor” [18]. In general, each comment was classified into two comment topic groups hierarchically. The first-level categories comprised methodology, clinical themes and other. Then, under each group, comment topics were further classified into subgroups, for example, clinical themes with a subcategory “clinical practice-related”. Topic categories are given in Table 1.

**Table 1:** Categories of comment topics

First-level topics	Second-level topics
	Study design
	Population
	Data
	Intervention
	Models
*Methodology*	Outcomes
	Results
	Analysis
	Explanation
	Generalizability
	No specific illustration (i.e. a few methodological limitations existed)
*Clinical themes*	
Biology	Biological mechanisms
	Genetic issues
Diagnosis	Diagnostic inconsistency
	Diagnostic difficulty
Treatment and drug	Alternative treatment
	Dosage issues
	Drug interactions
	Safety concerns
Medical evidence	Evidence integration
	Clinical evidence integration
	Clinical evidence-related
	Clinical practice-related
	Future studies needed to verify results
Other clinical issues	Animal model
	Ethical issues
	Evidence-based medicine
	Ignored comment point
	Implications
	Just mentioned
*Other*	Misinterpretation by readers
	Propose a new hypothesis
	Reference being retracted
	Infusion of politics into science
	Updated version

Once the comment topics were determined, we identified the sentiment orientation of the overall comment: supportive, critical or neutral. After going through the comments’ full texts in the above section, reviewers located the sentences with clear sentiment and then manually evaluated the document sentiment orientation (Table 2).

**Table 2:** Examples of comment sentences in published comments

Sentiment orientation	Topics	Comment sentences
Positive	Clinical practice-related	Our results are *remarkably similar to* those shown by Arshad et al.
Negative	Analysis	We read Skipper and colleagues’ article (1) with interest *but disagree with* some of their conclusions, which we believe could not be made from the data shown…
Neutral	Just mentioned	Lopinavir/ritonavir also seemed to be successful in treating a 62-year-old in Spain with COVID-19, but a recently published trial suggests the drug may not be effective

**Fig. 2:**
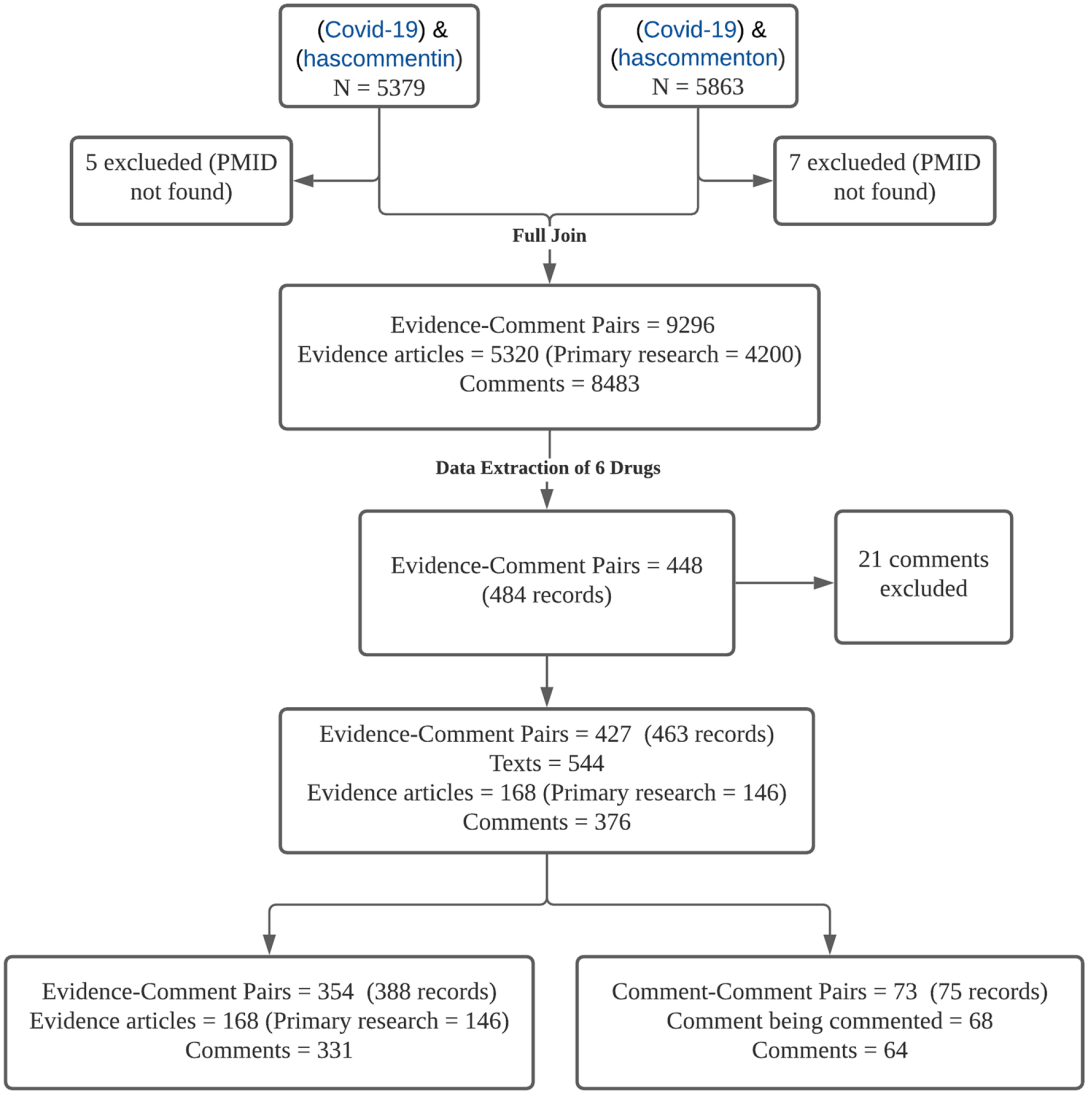
Data collection and preprocessing

After labelling all full-text articles, 21 comments were excluded because they were in Spanish and could not be translated accurately or because an evidence–comment pair was matched incorrectly in PubMed. Finally, 168 evidence articles (146 primary research articles; 22 other research articles) and 376 accompanying comments were included in this study. Two groups of pairs were included for a total of 427 pairs: evidence–comment pairs (354 pairs representing comments on primary research articles) and comment–comment pairs (73 pairs representing comments on previous comments). In this study, we analysed both groups and used “evidence–comment” to refer to all of them. Figure 2 shows the data collection and preprocessing procedure.

### Data analysis and visualization

Cytoscape 3.9.0 software was utilized to draw evidence–comment networks (ECNs). In an ECN, each publication (identified by their PubMed ID, or PMID) is represented as a node. Edges represent the relations of evidence–comment pairs, with the direction of the edge pointing from one comment publication to the article on which it comments.

For each drug, the largest connected subgraph was analysed in depth for elaborating comment-driven evidence assertions. Specifically, first, each primary article was manually reviewed for a research claim. Next, comments for each article were read to identify the comment sentiments (positive/negative/neutral) and comment points (e.g. methodology, biological mechanism) towards the given claim. For example, if a randomized controlled trial (RCT) research claim that “HCQ is effective on COVID-19” received five critical comments, then a comment-driven assertion here would be that “HCQ is effective on COVID-19 is negated”. Such assertions indicated the evidence appraisal results by leveraging the rich information provided by comments towards the primary evidence. Third, at the subgraph level, all appraised evidence results were aggregated to conclude an integrated assertion on the specific topic. Comment-driven evidence assertions of each drug’s largest one or two subgraphs were then summarized and compared with the final recommendations in the WHO guidelines.

## Results

### Overall ECN analysis

**Fig. 3:**
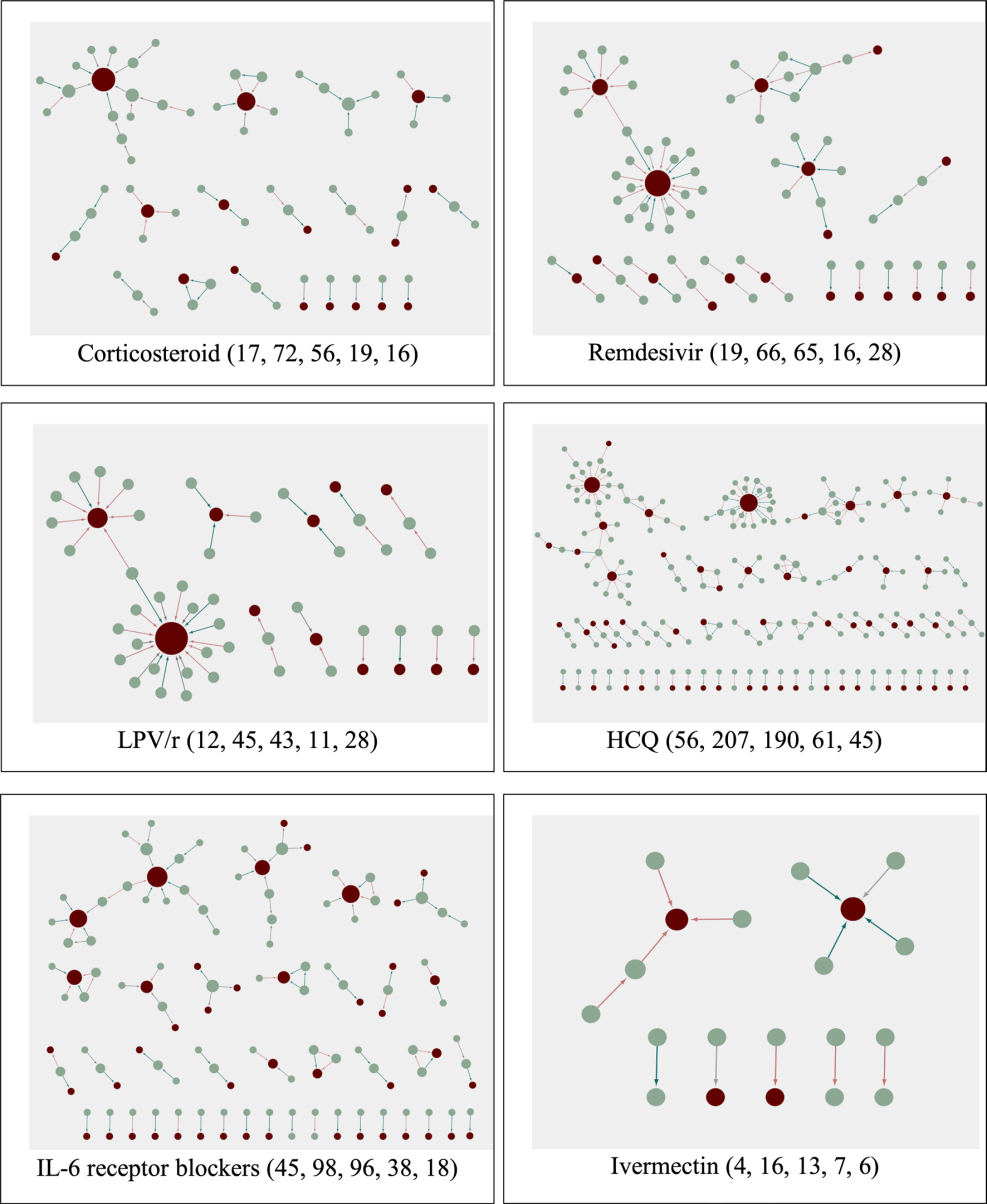
Overall drug evidence–comment networks. For each drug, evidence count, comment count, evidence–comment pair count, the count of subgraphs and the count of nodes of the largest subgraph are listed in sequence. The red node indicates original research, and the green node indicates secondary research (i.e. review) or comments. Green arrows represent supportive comments, red arrows represent critical comments, and grey arrows represent neutral sentiment. The larger the node in all connected component networks in each subgraph, the higher the degree of centrality

Overall directed ECNs of all six drugs were drawn as shown in Fig. 3.

**Fig. 4:**
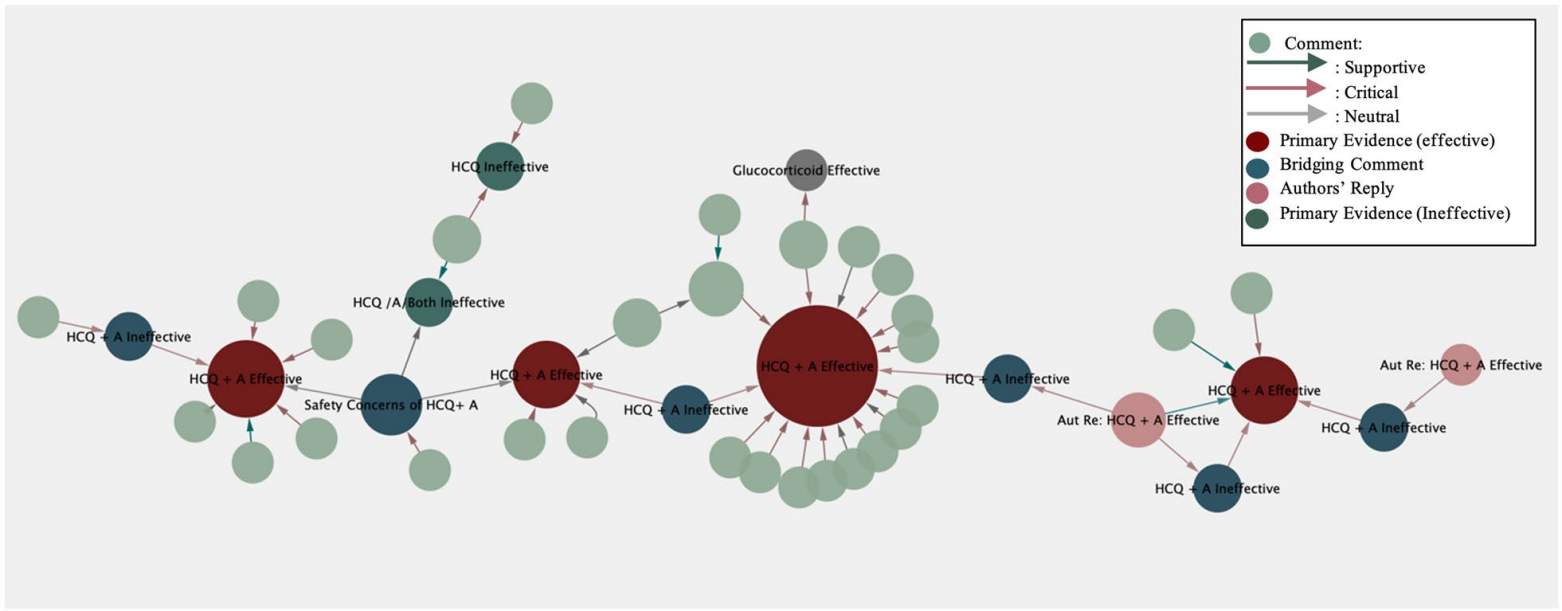
Treatment efficacy of HCQ + A on COVID-19 was negated

For example, in Fig. 4, all four HCQ + azithromycin (A) early research articles concluded that HCQ + A was effective for COVID-19; in particular, Gautret’s team published two articles to demonstrate this view [24–27]. By contrast, significant bridging reviews either challenged the efficacy of this combination on COVID-19 or expressed safety concerns of this off-label usage, except for two authors’ replies to defend their positions [28–33]. Furthermore, over two thirds of comments (75.6%) criticized (red arrows) those articles claiming treatment effectiveness, such as case report evidence of inefficacy and concerns with this treatment [28, 30, 34].

Significantly, when Alizargar commented on two articles claiming the efficacy of HCQ + A, he also commented on Rosenberg et al.’s research, which found no evidence of efficacy for either HCQ or A or the combination of both (HCQ/A/both), to integrate conflicting studies and evidence [28, 35]. In this way, the commenter completed evidence appraisal by criticizing problematic evidence and providing related evidence to strengthen their own position and thus connected relevant evidence forming an ECN. In the end, an HCQ + A treatment knowledge path was achieved from “efficacy found” to “efficacy negated” due to comments challenging these viewpoints. This was consistent with the third version of the WHO guideline on 17 December 2020, which recommended against using HCQ on COVID-19, regardless of the severity of disease [36].

A relevant absolute conclusion was not reached from the above ECN. However, a more conclusive claim could be developed as more evidence–comment pairs joined together with consistent sentiments. In Fig. 4, the general consensus on the efficacy of HCQ + A for COVID-19 is negative.

Except for LPV/r, the detected effectiveness from the top subgraphs of the five drugs (remdesivir, HCQ, ivermectin, corticosteroids, IL-6 receptor blockers) were consistent with the respective WHO guidelines. Specifically, for HCQ and tocilizumab (IL-6 receptor blockers), we looked at the largest subgraph, and for the other four drugs we went through the largest two subgraphs. For a more in-depth analysis of the ECNs for the other five drugs, see Additional file 1.

Comments can also reflect uncertainties in evidence. Research commentaries may publish original research and case reports that may be insufficient to produce an original research article but cannot be neglected as vital evidence to supplement the field literature [37]. In the case of IL-6 receptor blockers, Hassoun et al.’s concern regarding the uncertainty of the ideal dose of tocilizumab was confirmed by WHO’s latest guideline [23, 38]. This suggests that comments perform evidence appraisal to promote the certainty of knowledge by resolving uncertainty as well as highlighting uncertainty.

### Comment sentiment and topic analysis

After a detailed analysis of the largest two subgraphs, the overall sentiment orientation for all the subgraphs was computed for each drug. The overall sentiment could help further identify the overall effectiveness propensity of each drug for researchers and clinicians. We found that the overall sentiment orientation fully aligned with the recommendation propensity of WHO guidelines.

### Overall sentiment analysis

We calculated the overall sentiment in the commentaries for each drug group. Without considering specific topics that were discussed (i.e. treatment efficacy, inefficacy or mechanisms of action) and only considering the distribution of comment sentiment orientations, we found that the results were aligned with recommendations in WHO guidelines. Specifically, WHO guidelines recommended using IL-6 receptor blockers (tocilizumab/sarilumab) and corticosteroids for patients with severe or critical COVID-19. Only IL-6 receptor blockers (48; 25) and corticosteroids (28; 12) received more supportive comments than critical comments (Table 3). HCQ (62; 94), remdesivir (19; 30), LPV/r (9; 25) and ivermectin (4; 7) received fewer supportive comments than critical comments.

**Table 3:** Sentiment orientation of comments for six drugs

Drugs	WHO guideline date	Recommendation sentences	Recommend using?	Sentiment orientations (%)		
				Positive	Negative	Neutral
Corticosteroids	2 September 2020 (Version 1)	“A strong recommendation for systemic corticosteroid (severe patients)” “A conditional recommendation against corticosteroid (non-severe patients)”	Yes	28 (50.00%)	12 (21.43%)	16 (28.57%)
IL-6	6 July 2021 (Version 5)	“A strong recommendation to use IL-6 receptor blockers (tocilizumab or sarilumab) in severe or critical patients”	Yes	48 (50.00%)	25 (26.04%)	23 (23.96%)
HCQ	17 December 2020 (Version 3)	“A strong recommendation against the use of hydroxychloroquine”	No	62 (32.63%)	94 (49.47%)	34 (17.89%)
Remdesivir	20 November 2020 (Version 2)	“A conditional recommendation against the use of remdesivir for patients with COVID-19”	No	19 (29.23%)	30 (46.15%)	16 (24.62%)
LPV/r	17 December 2020 (Version 3)	“A strong recommendation against the use of lopinavir/ritonavir”	No	9 (20.93%)	25 (58.14%)	9 (20.93%)
Ivermectin	31 March 2021 (Version 4)	“A strong recommendation to use IL-6 receptor blockers (tocilizumab or sarilumab) in severe or critical patients”	No	4 (30.77%)	7 (53.85%)	2 (15.38%)

Besides sentiment orientation, comments also indicate the applicability of drug usage at different levels of severity of disease (non-severe, severe, critical). These were consistent with the conditional recommendations for/against the use of the drug (out of six drugs, three drugs have conditional recommendations) in WHO guidelines, suggesting the reliability of comment-driven evidence assertion. Detailed evidence and comment examples for these three drugs are listed below:

- *Corticosteroids*: Both original research articles and commentaries include conditional recommendations for corticosteroids. A meta-analysis concluded that “*[p]atients with severe conditions are more likely to require corticosteroids*”, which was further commented on [39]. An article focused on the rehabilitation of COVID-19 without mentioning corticosteroids received a comment stating that “*[t]he goal of management for these critically ill patients should… Continuous realignment of care goals for these patients including short and rational use of corticosteroids, low dose…”* [40].
- *Remdesivir:* A study concluding the benefits of remdesivir on hospitalized COVID-19 patients [41] received a comment highlighting that “*the benefit of remdesivir was limited to patients who received oxygen therapy; it did not extend to those with mild disease or those who were receiving advanced ventilation*” [42]. This may suggest the benefits of remdesivir for severe cases rather than critical cases, which implies an alignment between insights from comments and recommendations from guidelines.
- *IL-6 receptor blockers*: An article on the treatment of 21 patients concluded that “*[p]reliminary data show that tocilizumab, which improved the clinical outcome immediately in severe and critical COVID-19 patients, is an effective treatment to reduce mortality*”, and received five comments [43]. A comment on two studies on the efficacy of tocilizumab in severe or critical cases [44, 45] served to complement their nine cases (eight were admitted or transferred to the intensive care unit) with presumed cytokine release syndrome (CRS) and COVID-19: “*while administration of tocilizumab in patients with COVID-19 exhibiting signs of CRS appeared to show clinical improvement, the ideal setting and dose of administration requires further study*” [38].

### Comment topics

To probe the coverage of comments, we compared the comment topics with the concerns of WHO guidelines and other evidence appraisal systems. We aimed to uncover whether comment topics were in accord with the core concerns of evidence appraisal criteria in the development of clinical practice guidelines and whether they went beyond these topics.

The quality of evidence, especially the research methodology, is the most crucial factor in developing guidelines and recommendations. Nevertheless, having high-quality evidence alone does not entail a strong recommendation. Other factors affecting recommendations include clinical applications (most significantly, the benefits and risks of an intervention), patient values and preferences, and costs, among other factors. [46, 47]

**Fig. 5:**
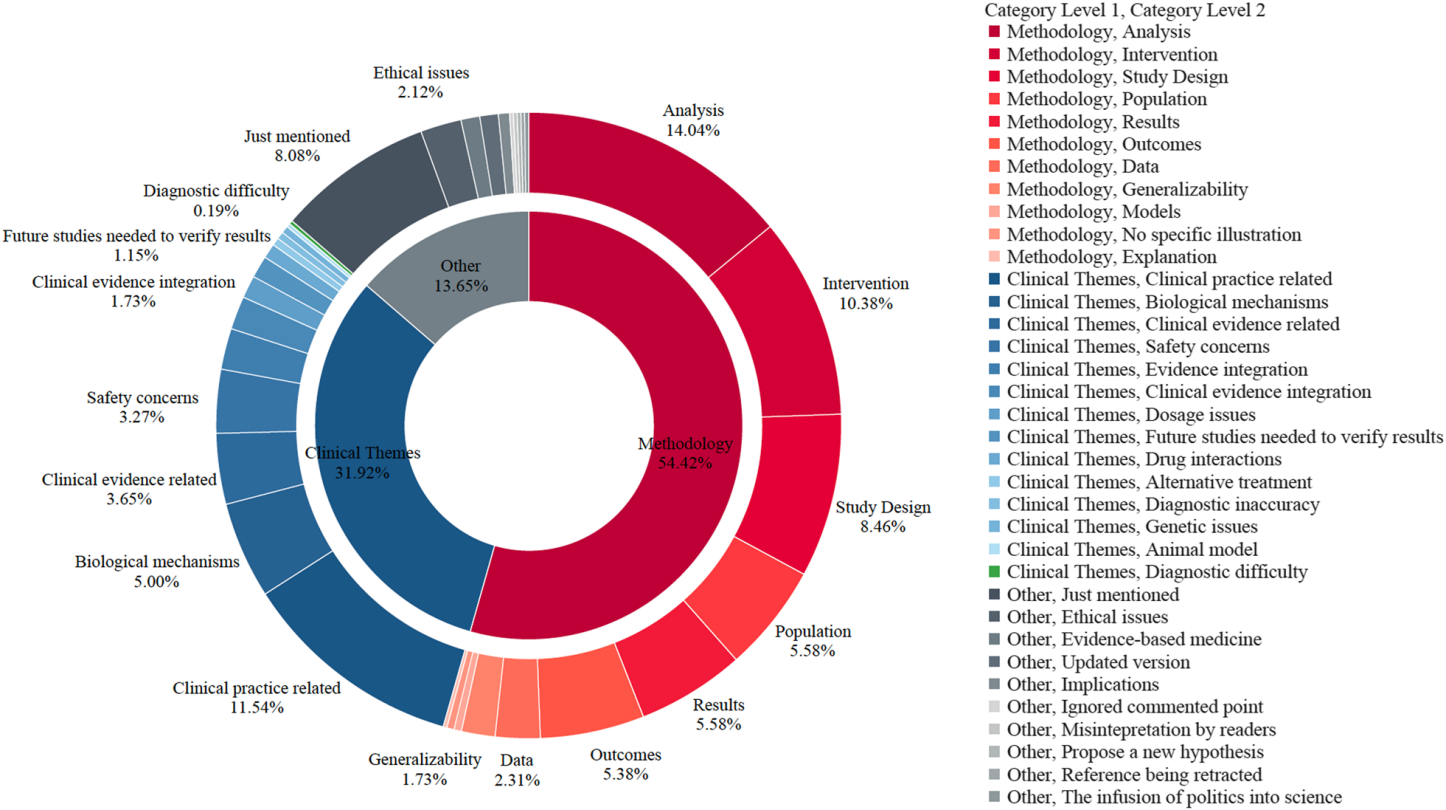
The overall distribution of comment topics. The chart includes two layers, and the inner layer is the first level of comment topics. The outside layer is the second-level topic of each comment under the first level

The distribution of comment topics is plotted in Fig. 5. The overall distribution showed that the leading comment topic was methodology (54.42%), followed by clinical themes (31.92%) and other (13.65%). These results aligned with Kastner et al.’s findings [18]. Methodology topics covered the overall research process, including study design, population, data, intervention, models, outcomes, results, analysis, discussion and generalizability. In clinical themes, topics covered biology, diagnosis, treatment and drug, medical evidence and other clinical issues. In the Other category, topics consisted of ethical issues, new hypotheses, knowledge clarification and other issues.

Specifically, the top three subcategories of methodology topics were analysis (14.04%), intervention (10.38%) and study design (8.46%); in clinical theme topics, these were clinical practice-related (11.54%), biological mechanisms (5.00%) and clinical evidence-related (3.65%); in other topics, these included just mentioned (8.08%), ethical issues (2.12%) and evidence-based medicine or experience-based medicine (0.96%).

As the results revealed, the points that commentaries focused on were in accord with the concerns of current grading systems (i.e. Grading of Recommendations Assessment, Development and Evaluation [GRADE]). As an overall clinical evidence appraisal system, GRADE mainly focuses on two aspects, namely factors that impact evidence quality (study design) and factors that impact the strength of recommendations, which are both linked to comment topics [47]. When evaluating evidence quality, it considers factors influencing the overall study design (i.e. risk of bias, imprecision, inconsistency), which correspond to methodology comment topics (i.e. population, study design, outcomes). When evaluating the strength of recommendations, GRADE focuses on patient values and pros and cons (i.e. the balance of benefits and harms, benefits and costs of resources), which can be mapped to clinical themes and the other groups, such as safety concerns and ethical issues. This match further shows the close association between comment topics and concerns of grading guidelines. Table S2 describes how comments address GRADE subdomains (Additional file 1).

Though strongly related, GRADE concentrates on RCT or other observational trials for the certainty of evidence. Comments can contribute to various aspects of decision-making beyond science, such as politics, economics, availability and feasibility. In the “other” category of our comment topics, especially in the subcategory of “just mentioned”, commentators mention the research evidence without detailed appraisal but discuss other related topics. For example, Self et al. [48] demonstrated that HCQ was ineffective based on their RCT, and Saag commented on this article not to appraise evidence quality but to criticize the infusion of politics into science resulting in the research craze of HCQ, despite the lack of benefits detected [49].

### Efficiency of comment-driven evidence appraisal

Consistency and coverage are prerequisites to enabling comment-driven evidence appraisal to aid clinical policymaking. However, what makes it the most competitive is the timeliness. We analysed the comment time span for each drug. Specifically, we extracted the date of the first published critical comment for each drug and the first half of critical comments published compared to the date of WHO guidelines publication, in order to determine to what extent assertions shaped by critical comments are faster than the final released recommendations in WHO guidelines.

**Fig. 6:**
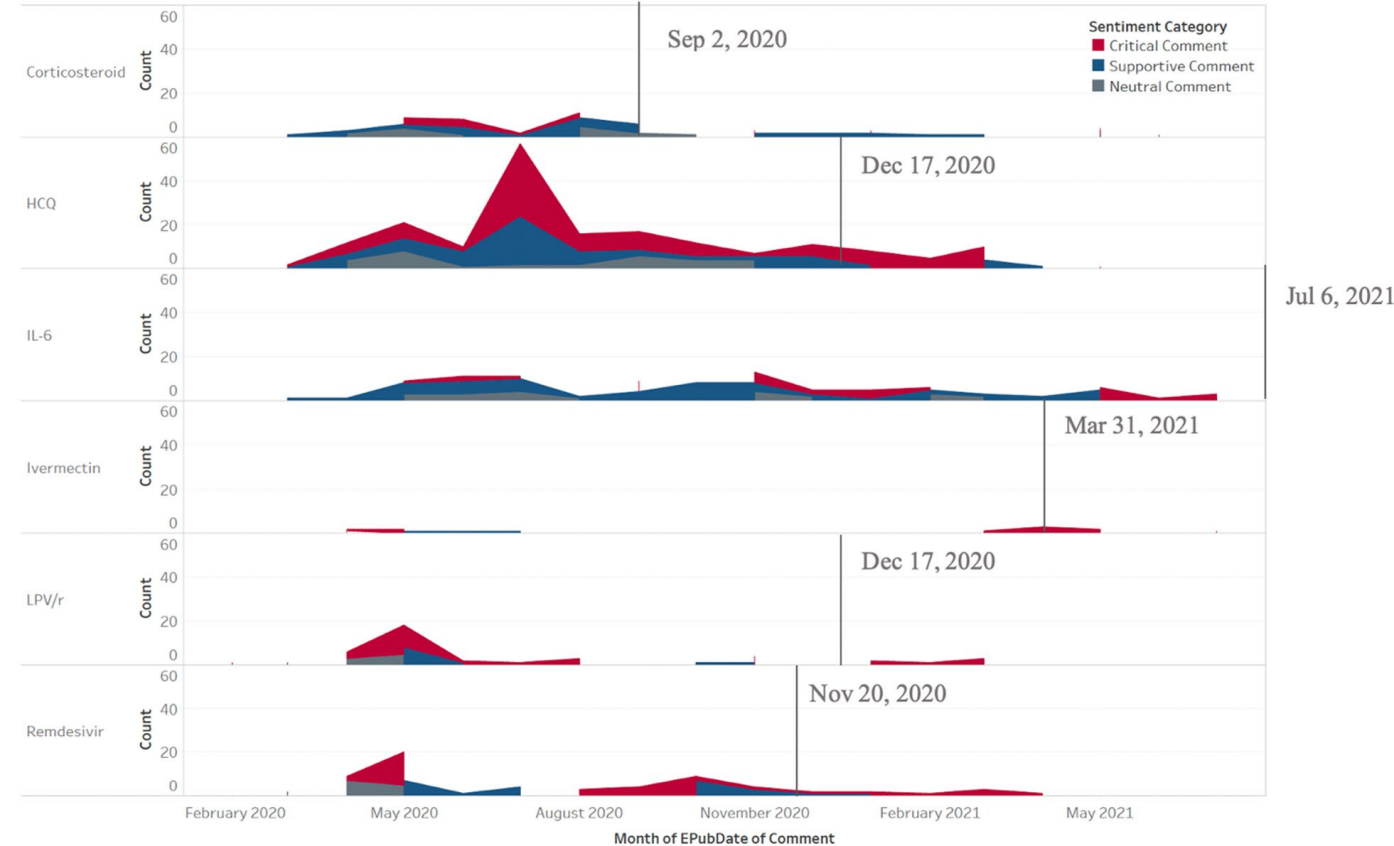
Time span of sentiment orientations for each drug

We plotted the comment time span of each sentiment orientation for each drug, as shown in Fig. 6. Red sections indicate the time span of critical comments. For each drug, the first critical comment (red section) emerged earlier than the publication date of WHO guidelines, by an average of 8.8 months. For each drug, the month that half of the negative comments had accumulated was determined: (1) June and July 2020 (corticosteroids, 2.5 months earlier), (2) May and August 2020 (remdesivir, 4.5 months earlier), (3) July and August 2020 (HCQ, 4.5 months earlier), (4) May 2020 (LPV/r, 7 months earlier), (5) April 2021 (ivermectin, 1 month later) and (6) November 2020 (IL-6 receptor blockers, 8 months earlier), respectively. On average this was 4.25 months earlier than the WHO guidelines. As for the query date of 21 July 2021, 6.03 months on average after guideline release, the first negative comments emerged much earlier, and the potential values inside could be mined for early critical clues for evidence appraisal.

Interestingly, for corticosteroids and IL-6 receptor blockers, all critical comments happened before the publication of guideline recommendations, which shows the acceptance of these two drug candidates. Since corticosteroids and IL-6 receptor blockers both fight virus-associated cytokine release syndrome in severe or critical cases instead of directly suppressing viral replication, as the safety concerns are solved, the controversy may gradually dissipate. By contrast, HCQ and remdesivir, two controversial candidates, continued receiving new critical comments even after the recommendations against their use in the WHO guidelines.

If an informatics approach could help detect critical signs or assertions faster, it would provide strong clues for clinical guideline development. Furthermore, the timeliness of the comment-driven methodology makes it a powerful approach for evidence appraisal when fast decisions are needed in urgent situations.

## Discussion

The present study validated how research commentaries can appraise clinical evidence and impact the shaping of knowledge, focusing on COVID-19 and six well-known drugs that have been used to treat it. Our results revealed the effectiveness of the largest subgraphs of five drugs (the exception being LPV/r) derived from relevant comments for predicting the subsequent recommendation in the WHO guidelines. The overall sentiment orientations derived from comments for each drug were fully aligned with guidelines, showing the consistency of comment sentiment. Further, comment coverage analysis revealed that methodology, clinical themes and ethical issues are core topics discussed in comments. This was well matched with the core concerns of WHO in their guideline recommendations and even went beyond these, including political and societal issues. Finally, for efficiency analysis, half of the critical comments appeared on average 4.25 months earlier than the release of the guidelines, which makes comment-driven assertions a timely appraisal tool. For clinical research, the median and mean time from publication of an article to the publication of a comment is 4 months and 6 months, respectively [14].

Generally, a short time window of the formal publication between evidence and comment adapts to one of the essential components—keeping updated—in the development of living guidelines. Compared to 2–3 years required to develop traditional guidelines [50], the emergence of rapid living guidelines has significantly accelerated the development cycle of clinical guidelines—for example, 3–4 months (median) for Australian national guidelines for treating stroke, and only 20 days (median) for Australian COVID-19 living guidelines incorporating new evidence [8]. In addition, a previous study revealed that the expected update frequency of guidelines for stroke treatment in Australia varied widely from 3-monthly (25%) and 6-monthly (23%) to yearly (30%) [51]. Although the update time of living guidelines is topic-dependent, the 4-month median time of published comments could fall into the updated time range of living guidelines. These timely comments on a variety of clinically relevant topics (e.g. drug interactions, related clinical practice, alternative treatment, case series reports) could provide significant information in various types of evidence appraisal supporting living guidelines updates, especially in emergency scenarios with only uncertain evidence. All the above results suggest that ECNs, including comment topics and sentiment orientation, can serve as supporting tools for evidence appraisal, detecting significant evidence and alerting for potential risks.

Scientific commentaries are an important method of scholarly communication, but they remain underutilized. Horton firstly pointed out that “failure to recognize the critical footprint of primary research weakens the validity of guidelines and distorts clinical knowledge” [15]. He stressed the important contribution of research commentaries in shaping clinical knowledge, especially criticism, which influenced our study [15]. More recently, Sahin et al. argued the significant role that commentaries play in evidence appraisal [13]. Our study lends support to their assertions through a systematic and quantitative analysis.

How to take advantage of this support tool in a simple but powerful way becomes a worthy discussion point. Here, based on our research findings, we suggest a comment-based appraisal framework based on the comment topics and sentiment orientations, as shown in Table 4.

**Table 4:** A comment-based evidence appraisal framework

Comment-based evidence appraisal	
Comment themes	Comment sentiment
Methodology	Supportive
Clinical applicability	Critical
Other	Neutral

For a given article, each comment would be analysed to identify its comment topics and comment sentiment. Under a specific comment theme, all sentiment orientations would be synthesized for a cumulative score. All scores of all themes would then be further combined to obtain the final assessment score. For different scientific questions, comment themes of interest could be filtered in producing the most relevant theme scope and weights, to help prioritize the most relevant comment topics.

Based on the proposed framework, automatic identification of comment topics and sentiments for evidence appraisal automation would also be essential in future work. Therefore, to take advantage of commentaries for evidence appraisal, we suggest that medical journals label comment sentiment and topics once comments are accepted for publishing. This could help create structured data for calculating comment-driven evidence assertions. Sahin et al. expressed a similar view in *The Lancet* by using controlled vocabulary, such as “lacks equipoise”, to represent journal articles (comments) [13]. This could be used to build a knowledge base to connect clinical studies and their comments in a structured format to leverage the potential of scientific commentaries in evidence appraisal. Natural language processing approaches can also be used to support this framework, especially the automatic identification of disease names (i.e. named entity recognition) and claim extraction research (i.e. rule-based and machine learning methods). Sentiment analysis techniques [52] (including the more specific citation sentiment classification [53, 54]) as well as topic modelling and classification methods [55] can be developed to assist these tasks.

Our study has some limitations. Firstly, we only included six drugs and one WHO baseline living guideline in this study to show the feasibility of the approach. Considering the small sample and the inconsistency among guidelines of different countries, the generalizability of this approach could be further validated by including more drugs and more guidelines in the analysis. Second, although crucial information of specific subgroups or phenotypes are involved in comments, we did not represent COVID-19 at finer granularity regarding the severity of the disease in annotation. Thus, the overall comment sentiment was towards COVID-19 as a whole rather than a specific severity and failed to align with the conditional recommendation for severe cases in the current version of the guideline [56]. Third, we annotated the comment sentiment towards an article rather than a claim. An article-level sentiment reflects whether a comment agrees or disagrees with the results of a study (may be effective or ineffective). A claim-level sentiment reflects whether a comment agrees or disagrees with a claim (i.e. remdesivir is effective). Thus, a fine-grained sentiment analysis for specific claims (i.e. aspect-based sentiment analysis) could more precisely reflect the assessment regarding a given assertion. Fourth, we did not consider whether there were conflicts of interest in the comments. If a comment made is on behalf of a particular stakeholder, its influence in evidence appraisal should be low due to a potential loss of objectivity. Fifth, manual sentiment annotation at the document level is complex and may contain some inconsistencies. Lastly, COVID-19 is a global concern with a large number of related commentaries published; for a disease with few commentaries released, the power and usability of this approach would be limited.

## Conclusions

Based on the consistency, coverage and efficiency performance, we conclude that research commentaries could be used to support evidence appraisal by providing clues that indicate the importance and validity of evidence to support evidence-based decision-making, especially in emergency situations like the COVID-19 pandemic. Scientific commentaries have a selection effect by appraising the benefits, limitations and other clinical practice issues of concern for existing evidence. It is notable that negative comments could provide a more detailed understanding regarding specific diseases/interventions not by only criticizing prior studies but also by introducing new viewpoints or evidence. Comments also have the potential to inform decision-making regarding both therapeutic efficacy and topics such as economics, politics and ethical issues, which are crucial aspects in health policy-making.

## Supplementary Information

**Additional file 1. **Additional materials.

**Additional file 2. **Adherence to reporting guidelines.

## Data Availability

The datasets generated and/or analysed during the current study are available from the corresponding author on reasonable request.
