# Supplementary material for: A comment-driven evidence appraisal approach to promoting research findings into practice when only uncertain evidence is available
Source: Health Res Policy Syst. 2023 Mar 27;21:25. doi: 10.1186/s12961-023-00969-9 (PMC10042414; doi:10.1186/s12961-023-00969-9)
Supplement: Supplementary file 1 — Additional file 1. Additional materials. [file 12961_2023_969_MOESM1_ESM.docx]

**Supplementary material**

**Section 1: Frequent concepts extracted using Pubtator Central**

We used the PubTator Central (PTC) text mining tool, which extracts concepts of CellLine, Chemical, DNAMutation, Disease, Gene, Genus, ProteinMutation, SNP, and Species from the title/abstract sentences of publications [1]. PTC is a high quality annotation tool that uses machine learning models with strong performance, from the F1 score of 83.10% [2] (TaggerOne [3] for cell lines) to 89.50% [4] (TaggerOne [3] for chemicals). In table S1, the top 5 concepts of disease, gene, species, chemical groups have at least 100 occurrences separately.

In general, the most common concepts are COVID-19-associated diseases, species, genes (infection mechanism), and drugs. With the persistent controversies around the effect of candidate drugs, we chose drugs as research objects for comment-driven evidence analysis and then we delved deeper for more drug candidates. We focused on whether the claims presented in the evidence are supported or refuted by comments, and how the methods and findings in the original articles are discussed in comments. Here, we used comment-driven evidence assertions to indicate the reshaped conclusions after the evidence was appraised and pruned by comments. The top 15 chemical-related concepts included 9 drugs: hydroxychloroquine, tocilizumab (IL-6 receptor blockers), remdesivir, vitamin d, azithromycin, chloroquine, lopinavir/ritonavir, steroids, aspirin. It is noteworthy that three widely discussed drugs during COVID-19—hydroxychloroquine, tocilizumab, and remdesivir—were detected among the top 5 drug concepts identified.

Table S1. Frequent concepts and counts of nine categories of entities

| Category | Concept | Counts |
| --- | --- | --- |
| Disease | covid-19 | 20213 |
|  | death | 3362 |
|  | infections | 2700 |
|  | neoplasms | 698 |
|  | respiratory distress syndrome | 635 |
| Gene | ace2 | 556 |
|  | c-reactive protein | 268 |
|  | il-6 | 212 |
|  | spike | 142 |
|  | ace | 131 |
| Species | patients | 17615 |
|  | sars-cov-2 | 5103 |
|  | coronavirus | 545 |
|  | sars-cov | 248 |
|  | mers-cov | 139 |
| Chemical | 25(oh)d | 614 |
|  | hydroxychloroquine | 496 |
|  | oxygen | 296 |
|  | tocilizumab | 258 |
|  | remdesivir | 186 |
|  | vitamin d | ﻿185 |
|  | azithromycin | 140 |
|  | chloroquine | 97 |
|  | alcohol | 90 |
|  | lopinavir/ritonav﻿ir | 87 |
|  | heparin | 84 |
|  | glucose | 75 |
|  | steroids | 62 |
| DNAMutation | delta69 | 1 |
|  | c.2129_2132del | 1 |
|  | c.2383g>t | 1 |
| ProteinMutation | d614g | 21 |
|  | n439k | 6 |
|  | q498h | 3 |
|  | q493k | 3 |
|  | a475v | 1 |
| Genus | influenza | 80 |
| CellLine | vero e6 | 8 |
|  | e6 | 4 |
|  | mn | 4 |
|  | mcf | 2 |
|  | calu-3 | 2 |
| SNP | rs4588 | 3 |
|  | rs7041 | 3 |
|  | rs12252 | 2 |
|  | rs11385942 | 1 |
|  | rs657152 | 1 |

**Section 2: GRADE subdomains and comment themes**

Table S2. The comparison of GRADE [10] subdomains and comment themes

| 1. Factors impacting quality of evidence | Comment themes |
| --- | --- |
| 1.1. Certainty can be rated down for: | |
| Risk of bias | Study design |
| Imprecision | Data |
| Inconsistency | Population, Interventions, Outcomes, Analysis |
| Indirectness | Population, Interventions, Outcomes |
| Publication bias | Data, Generalizability |
| 1.2. Certainty can be rated up for: | |
| Large magnitude of effect | Results |
| Dose-response gradient | Dosage issues |
| All residual confounding would decrease magnitude of effect (in situations with an effect) | Population, Diagnostic inaccuracy, Diagnostic difficulty |
| 2. Factors impacting strength of recommendation | |
| Quality evidence | Study design |
| The balance of benefits vs. harms and burdens | Biological mechanism, Safety concerns, Drug interactions |
| Patient values | Outcomes, Safety concerns, Ethical issues |
| Benefits and costs of resources | Alternative treatment, Ethical issues |

**Section 3: The detailed stories reflected by evidence-comment network for each drug**

- 1. **Remdesivir**

The treatment effectiveness of remdesivir remained controversial based on its largest two subgraphs in Fig. S1. The left subgraph is the biggest subgraph of remdesivir. However, it was not about remdesivir itself. It showed a network discussing 1) the effectiveness of remdesivir and 2) the combination effectiveness of lopinavir + ritonavir on COVID-19, connected by a review-type comment about immunodeficiency. In this comment, Hammarström et al. compared the effects of potential antiviral drugs (i.e., Remdesivir) when illustrating immunodeficiency administration in the COVID-19 pandemic. This comparison thus brought these two drugs together. Here is Hammarström et al.’s comment sentences:

*“The antiviral drug remdesivir has shown some effects during compassionate use in patients with COVID-19; however, randomized, placebo-controlled clinical trials have yet to prove its value. Another combination of antiviral drugs (lopinavir-ritonavir) did not provide any benefit for hospitalized patients with COVID-19 with severe disease in a randomized, controlled, open-label trial.”*


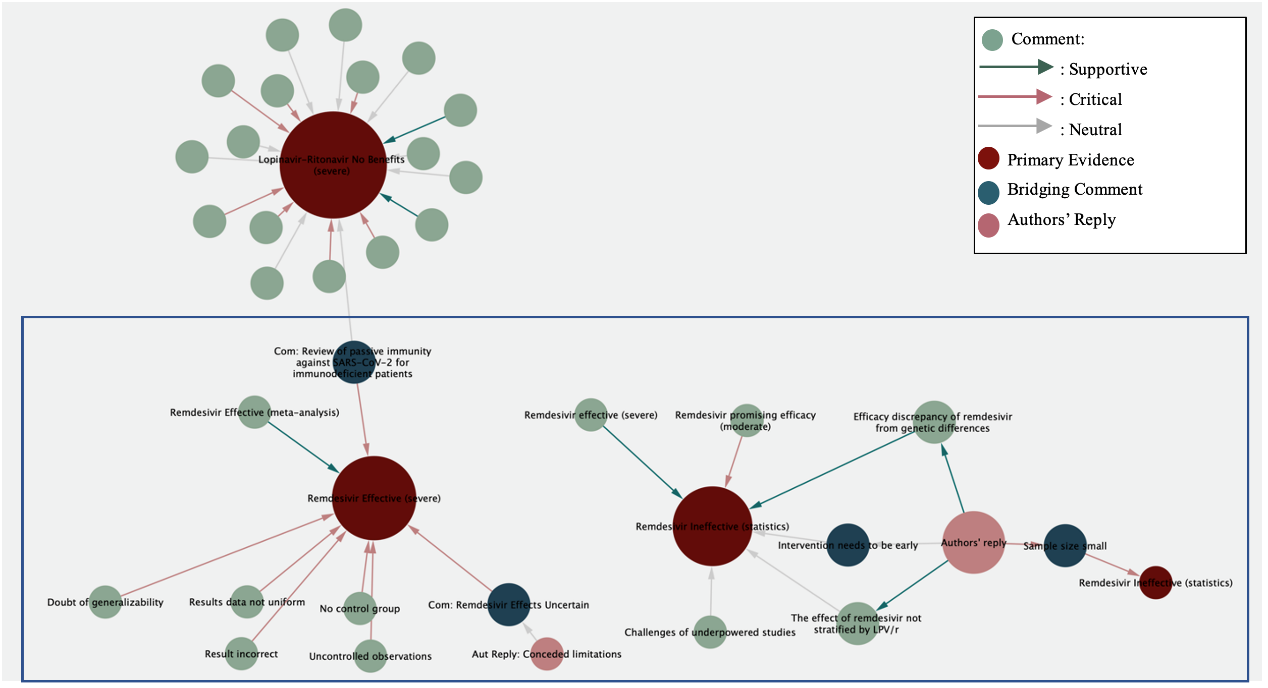


**Fig. S1. The effectiveness of remdesivir on COVID-19 was uncertain**

In this evidence-comment network, the comment from Hammarström et al. connected Grein et al.’s position that Remdesivir was effective on severe COVID-19 patients by its compassionate use in 61 patients, to the oppositive position from Cao et al. that no benefits were found with lopinavir-ritonavir treatment after a 199 patients’ trial [5, 6]. This connection introduced both supportive and refuting evidence of the efficacy of antiviral drugs to help integrate directly conflicting clinical evidence. Also, Hammarström et al. mentioned that no RCT had proved Grein et al.’s results of the efficacy of remdesivir [5, 7].

Further, when we focus on the analysis of remdesivir on the left below, the assertion that remdesivir was effective is undermined and challenged. Grein et al.’s study (Remdesivir, effective) received 7 criticisms out of 8 comments. Among these criticisms, researchers criticized therapy duration as unclear, doubted the generalizability and incorrect higher cumulative incidence of clinical improvement, and the most apparent problem of no control group was mentioned [8-13]. Grein et al. replied to Wu, Wu, & Lai’s “no control group” comment by conceding the limitations of their study and stressed that the results should be interpreted with caution since they were under particular compassionate use.

Nevertheless, it is hard to conclude the effectiveness of remdesivir with this evidence-comment community based on scientific evidence from only one article. Thus, we further explore the second largest subgraph of remdesivir on the right side of Fig. 5, which revealed its uncertain effect on COVID-19. Wang et al. conducted an RCT and concluded that remdesivir was not associated with significant clinical improvements in time [14]. This RCT received more neutral comments (50%, 3/6). The only critical comment refuted Wang et al.’s claim and pointed out that remdesivir could decrease the time to recovery for patients with moderate COVID-19 based on the US National Institutes of Health Adaptive COVID-19 Treatment Trial (ACTT) [15, 16].

Based on the leading two subgraphs of remdesivir, there are more critical comments than supportive ones on the effectiveness of remdesivir, which undermines its use for COVID-19, though uncertainties remained. The third version of WHO guidelines on 17 Dec 2020 recommended against using remdesivir and lopinavir for COVID-19, regardless of the severity of the disease. The concrete recommendation here echoes the result of comment-driven evidence assertion [17].

- 1. **Lopinavir/Ritonavir**

We extracted the top two subgraphs of Lopinavir/Ritonavir (LPV/r) combination to analyze its treatment effect as Fig. S2. As the above section illustrated, the largest subgraph of LPV/r was the discussion of the effectiveness of antivirals. On the left above component about LPV/r, Cao et al. declared no benefits observed in patients with severe COVID-19 from their RCT, and compared to supportive (11.1 %, 2/18) or critical (38.89 %, 7/18) comments, comments were more neutral (55.6%, 10/18) about the “no benefits” finding [6].

**
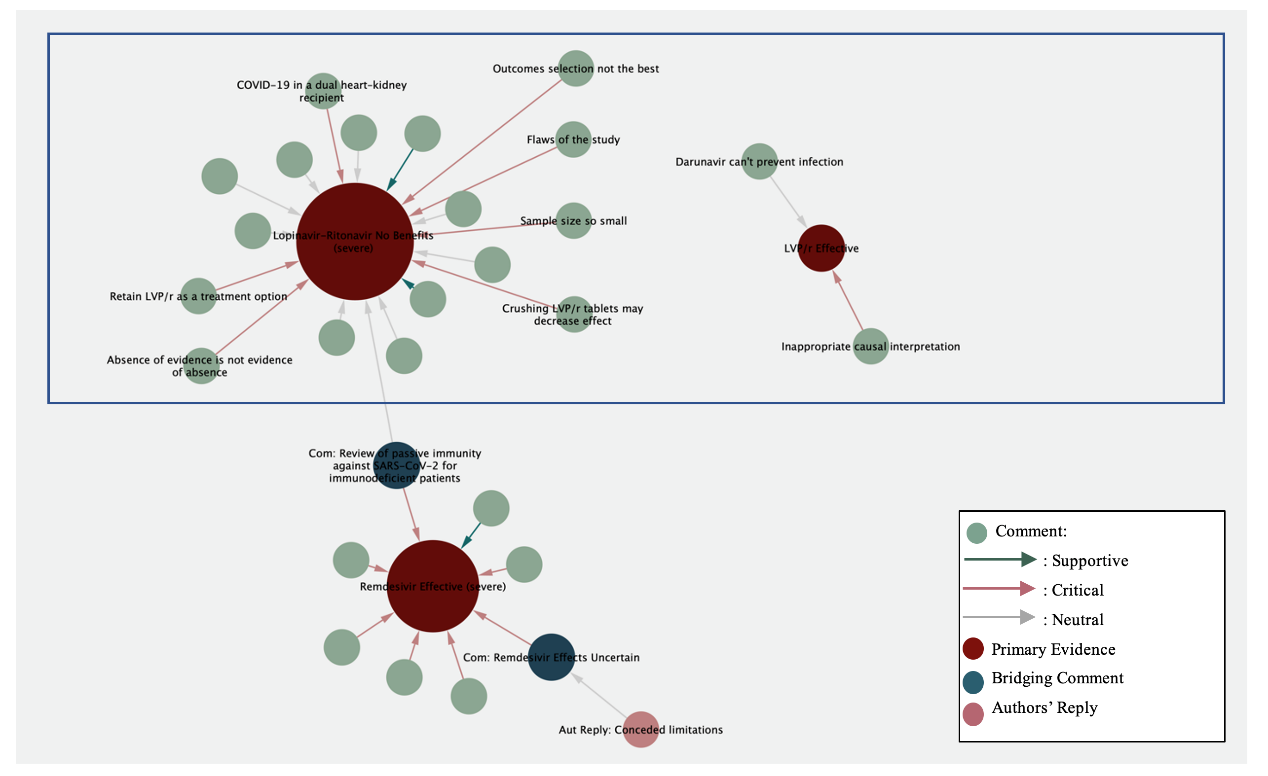
Fig. S2. Treatment efficacy of LPV/r on COVID-19 was uncertain**

Among negative comments, a series of methodology errors were judged, such as the “sample size is so small”, “crushing LPV/r tablets may weaken effect”, “primary endpoint (speed to symptom resolution) may not be the best”, and “late therapy and population selection bias” [18-21].

In addition, it is worthy to note that besides pointing out the weakness of this trial, critical comments emphasized the importance of not shutting the door on LPV/r RCT so early since “no benefits observed” does not mean inefficacy and “absence of evidence is not evidence of absence”, declaring incremental clinical improvements were crucial even without statistical significance at this urgent time [20-22]. Such calls reflected folks’ expectation for any possibility of hope and corresponded to the resumption of the WHO SOLIDARITY trial later [23].

Since only one article was included in this LPV/r research-comment community and no strong comment sentiments orientation detected, we looked more closely to the second subgraph on the right. Fortunately, evidence with strong comment sentiments orientation was found. Lim, Jeon [24] reported a case with significantly decreased β-coronavirus viral loads after LPV/r intervention (LPV/r, effective). This case report was criticized by Kim et al. for the inappropriate causal interpretation between laboratory results and therapeutic effects, regarding whether the virus decrease is a natural course or antiviral effect [25]. In addition, the other comment weakened this study and expressed that darunavir can not prevent SARS-CoV-2 infection in HIV patients based on their clinical practice related [26]. Lopinavir and darunavir are both HIV protease inhibitors and have similar structures. As a result, the second subgraph showed that the treatment of LPV/r was uncertain or even suspicious.

To sum up, comments of the top 2 largest subgraphs exhibited an overall neutral attitudes towards the efficacy of LPV/r (55%, 11/20), though strong negative evidence existed against this effectiveness. WHO guideline recommended against using HCQ and LPV/r on COVID-19, regardless of the severity of disease [17]. Though not fully aligned with the guideline, more negative comments already emerged as delved deeper. New evidence clues may reverse the assertion as keeping exploring. As we were exploring deeper, we were getting closer.

- 1. **Corticosteroid**

Corticosteroid has been suggested as a treatment candidate for COVID-19 due to its potent anti-inflammatory effects to mitigate the deleterious effects, such as lung injury and multisystem organ dysfunction, caused by the systemic inflammatory response of severe COVID-19 [27]. In contrast to direct antiviral effect, evidence on corticosteroid more focused on its safety and peripheral effects than virus-related efficacy. We analyzed the top two sub-graphs of corticosteroid in Fig. S3, as the largest sub-graph only contains three nodes discussing the efficacy of corticosteroid.


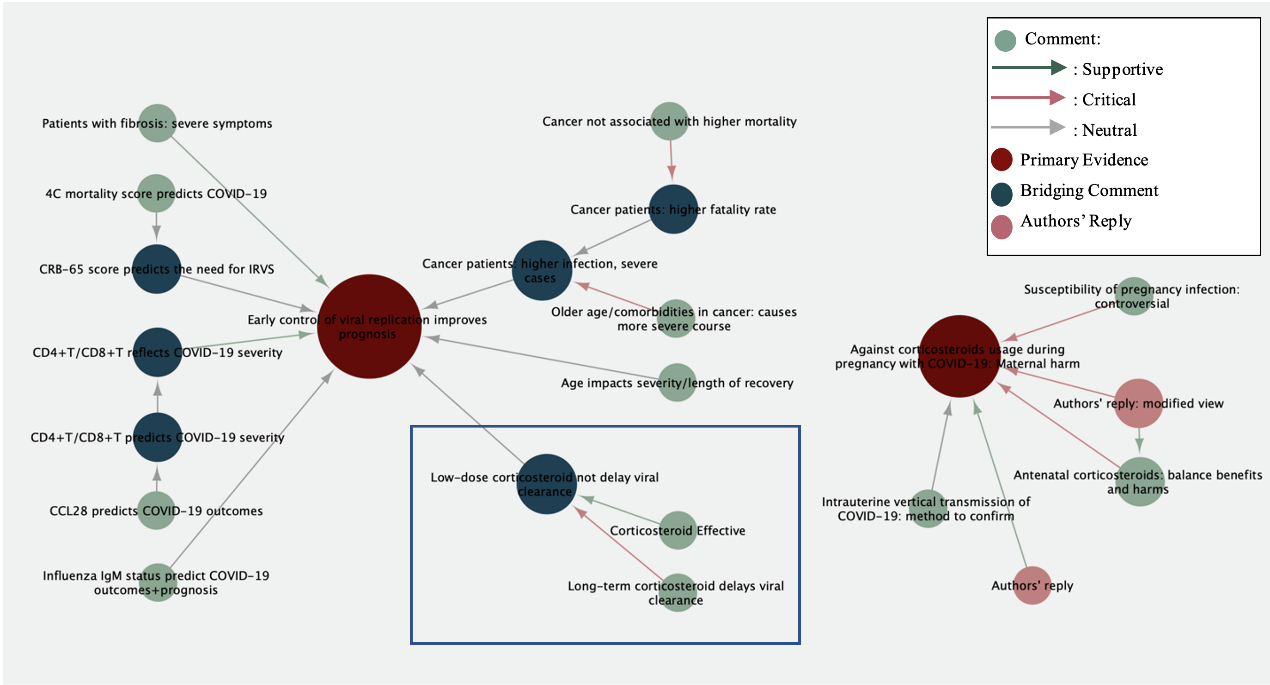


**Fig. S3. Treatment efficacy of corticosteroid on COVID-19**

Chen et al. discussed the clinical progression of COVID-19, contending that it was crucial to control viral replication in the early stage [28]. This is the original core research in the biggest subgraph involving corticosteroid. Comments of this article mainly discussed influential factors related to COVID-19. Among them, only one comment explored the treatment of COVID-19 and suggested that “low-dose of corticosteroid therapy may not delay viral clearance”, as fast viral clearance is an essential factor of recovery [29]. This suggested corticosteroid as a potential candidate for treating cytokine storm syndrome of patients with severe COVID-19, without the concern of the delay of viral clearance.

However, this commentary received two conflicting comments afterwards. Boglione et al. supported the research of Chen et al. by disclosing their own experience with 149 patients, finding that corticosteroid treatment on time has a positive effect on patients [30]. By contrast, Jung et al. disagreed with this comment and reported a case of a critically ill patient with COVID-19 in Korea detecting viral shedding delay after receiving low-dose corticosteroid, undermining the effect of corticosteroid [31]. It is hard to conclude the effect of corticosteroid on COVID-19 from this subgraph. Thus, we went through the second largest sub-graph for more evidence-comment information.

The positive effect of corticosteroid on COVID-19 was revealed from the 2^nd^ largest sub-graph. It began with an article about the antenatal administration of COVID-19 patients, against the antenatal corticosteroid therapy for fetal lung maturity during pregnancy of patients with COVID-19, considering deleterious effects corticosteroid on COVID-19 [32]. Two comments of this article discussed the usage of corticosteroid, including an authors’ reply. Liauw et al. firstly refuted the claims of maternal harm since the impact of corticosteroid in nonpregnant was unclear; secondly, they argued that the absolute benefits of antenatal corticosteroid vary per week since the baseline risks of neonatal morbidity decreased as gestational age increased [33]. Then, interestingly, the authors replied and modified their original view with the new evidence, recommended to use corticosteroid during pregnancy with the preterm risk [34, 35]. The reversion made the 2^nd^ sub-graph determined the benefits of corticosteroid on COVID-19. Here, as this comment provided new evidence to the original research evidence which reversed the initial assertion, comment-derived evidence created.

Based on the top 2 sub-graphs, the potential of corticosteroid on COVID-19 was detected, challenged, and further confirmed, and this may further evolve as new studies emerge. In addition, this finding corresponded to the first version of WHO guideline, which recommended to use corticosteroid on severe patients and conditionally recommended against using corticosteroid for non-severe patients. Corticosteroid was usually used to fight cytokine release syndrome (CRS), which would develop into severe or critically ill COVID-19 patients.

- 1. **Ivermectin**

The two largest sub-graphs of ivermectin were analyzed in detail, as shown in Fig. S4. As a result, the comment-driven conclusion from the two ECNs was that “ivermectin effective” was unsupported.


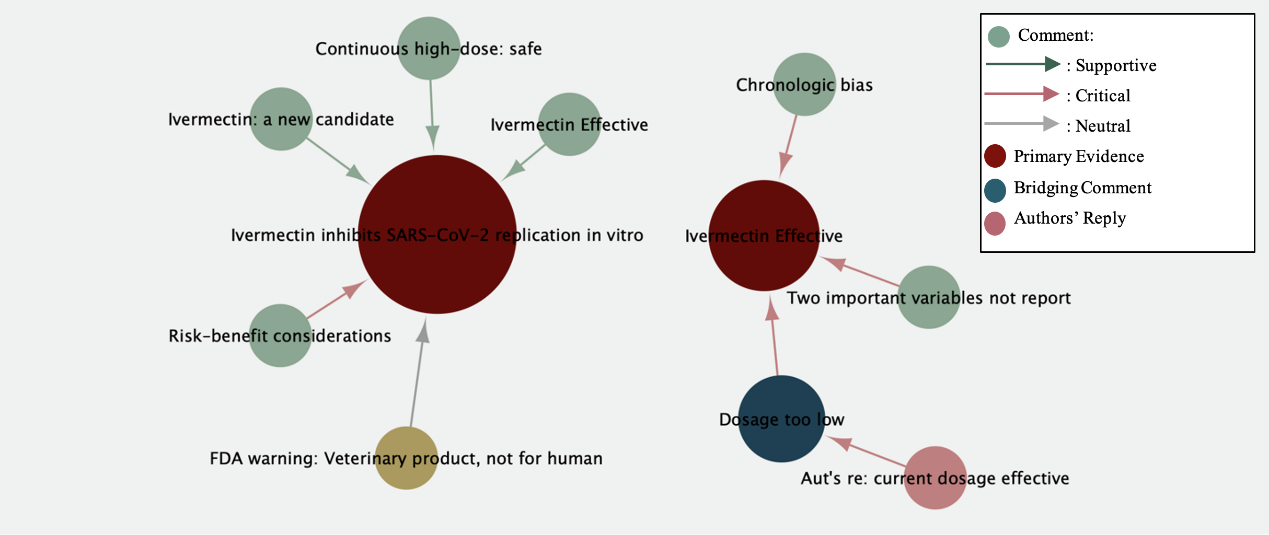


**Fig. S4.** **The treatment efficacy of ivermectin on COVID-19 was unsupported**

In the subgraph on the left, the seemingly good effectiveness of ivermectin was shown. Caly et al. stated that ivermectin could inhibit the replication of SARS-CoV-2 in vitro and concluded that it needs further research for possible benefits in humans cautiously [36]. This article received three supportive, one neutral comment, and one critical comment, which seemed promising from peers’ perspectives with more supports [37, 38] [39] [40, 41].

However, the neutral comment was from the chief editor of *Antiviral Research*, where Caly et al.’s work was published. Bray et al. asserted that this work had been widely spread online and ivermectin being incorrectly interpreted as a treatment drug, thus causing a warning from FDA that “ivermectin in veterinary products should not be used for human therapy” [37]. In sum, the effectiveness of ivermectin cannot be concluded from the left ECN. Moreover, the FDA warning emphasized that it was misleading to consider ivermectin as an effective human treatment.

The second biggest sub-graph displayed a comment-driven “ivermectin effective: questionable” assertion. Rajter et al. concluded that ivermectin was associated with lower mortality, especially in patients with severe pulmonary involvement [42]. This article received three critical comments, pointing out a series of methodology errors of the research, including chronology bias, low dosage intervention, not reporting two essential variables that may impact the results (time of symptom onset, patients’ health insurance coverage) [43-45]. With all comments negative, the comment-driven conclusion from the network was that the efficacy of ivermectin was unsupported.

Based on the top 2 sub-graphs of ivermectin above, the comment-driven assertion was that ivermectin was not practical for COVID-19. Correspondingly, WHO finally suggested not to use ivermectin for COVID-19 except for clinical trials in the 4^th^ version of the guideline [46]. This was a noteworthy finding. Even though considerable peers were positive, a warning sign could be detected from comments to trigger doubt about the validity of effectiveness of ivermectin. After combining to more sub-graphs, a reversed knowledge path of ivermectin would expectedly be detected.

- 1. **IL-6 receptor blockers** **(tocilizumab/****sarilumab)**

The first biggest sub-graph about IL-6 receptor blockers was deeply explored, showing that TCZ (IL-6 receptor blockers) was effective on severe COVID-19 patients in Fig. S5. This was consistent with the latest version of the WHO guideline, which highly recommended using IL-6 receptor blockers in severe patients [46].

**
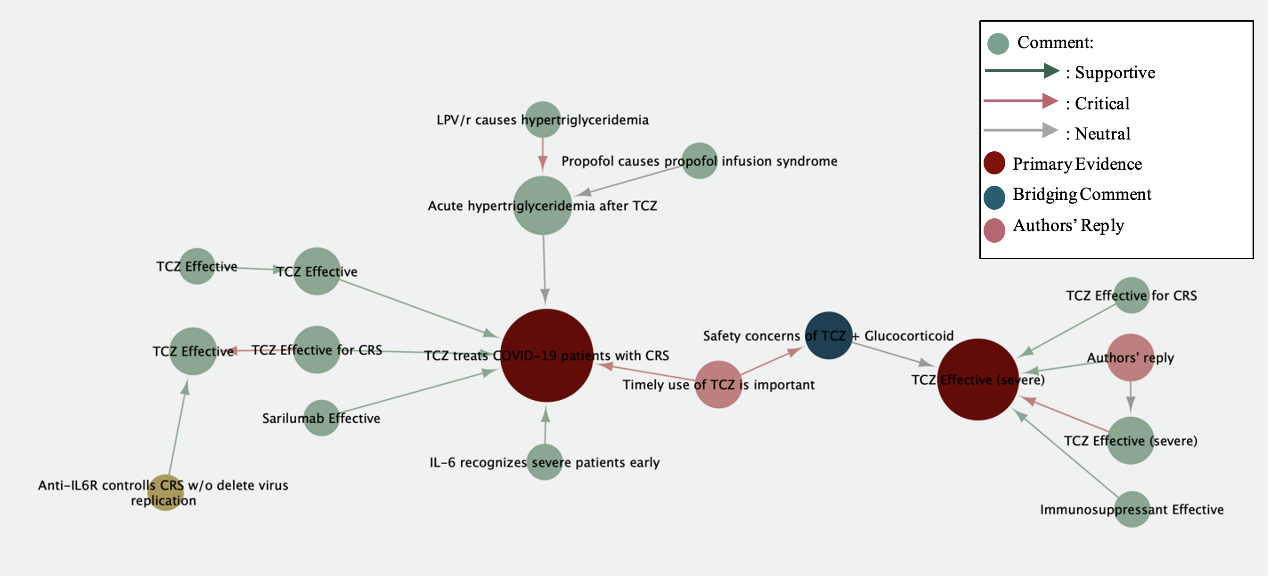
Fig. S5. TCZ (IL-6 receptor blockers) was effective on severe COVID-19**

Specifically, Xu et al. claimed that tocilizumab (TCZ) was effective in reducing mortality in severe COVID-19 patients, and this article was commented on by Yang et al. concerning the safety of the combined use of TCZ and glucocorticoids according to Luo et al.’s research that this combination resulted in disease aggravation [47-49]. Then Xu et al. replied to Yang et al.’s comment and commented on Luo et al.’s research at the same time because Yang et al. mentioned it, explaining Luo et al.’s worse clinical outcomes with the combination may be due to TCZ usage on patients’ late stages to reinforce the statement that TCZ was effective [48-50]. This back and forth discussion connected two relevant original studies and formed an evidence-comment network for the effectiveness of TCZ.

The majority of comments (N = 10) of these two original articles believed that TCZ/Sarilumab was effective [51-60]. Among them, three comments pointed out that TCZ was effective in cytokine release syndrome (CRS), an acute severe systemic inflammatory response resulting from the excessive synthesis of IL-6, which was associated with the SARS-CoV-2 infection [52, 53, 56]. Also, Buonaguro et al. suggested that the reason why TCZ treated COVID-19 was that it could control CRS instead of decreasing effects of virus replication [52].

Except comments about the effect of TCZ, Morrison et al.’s case report declared that they detected acute hypertriglyceridemia after TCZ usage on COVID-19 patients [61]. However, Sharma commented and supplemented that hypertriglyceridemia was associated with long-term administration of high-dose propofol in intubated COVID-19 patients, not TCZ [62, 63]. Besides, Hassoun et al.’s comment (case report) mentioned the uncertainty of the ideal dose of tocilizumab (IL-6 receptor blockers), confirmed by WHO’s latest guideline [46, 56] as shown in Fig. S6. This suggested that comments did evidence appraisal to promote the certainty of knowledge by clarifying (resolving) uncertainty as well as alerting (detecting) uncertainty.


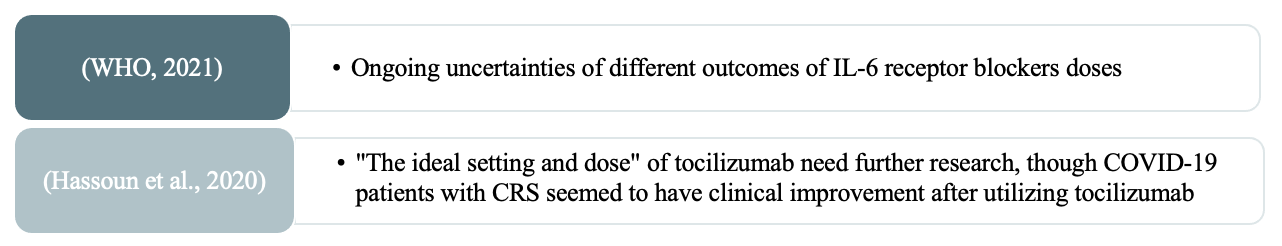


**Fig. S6. Uncertainty mentioned both in a comment and then in the 5^th^ version of WHO guideline**

**References**

1. Wei, C.-H., et al., *PubTator Central: Automated Concept Annotation for Biomedical Full Text Articles.* Nucleic Acids Res, 2019.

2. Arighi, C., et al., *Bio-ID track overview.* Proc. BioCreative Workshop, 2017: p. 376.

3. Leaman, R. and Z. Lu, *TaggerOne: joint named entity recognition and normalization with semi-Markov Models.* Bioinformatics, 2016. **32**(18): p. 2839-46.

4. Li, J., et al., *BioCreative V CDR task corpus: a resource for chemical disease relation extraction.* Database (Oxford), 2016. **2016**.

5. Grein, J., et al., *Compassionate Use of Remdesivir for Patients with Severe Covid-19.* N Engl J Med, 2020. **382**(24): p. 2327-2336.

6. Cao, B., et al., *A Trial of Lopinavir-Ritonavir in Adults Hospitalized with Severe Covid-19.* N Engl J Med, 2020. **382**(19): p. 1787-1799.

7. Hammarström, L., et al., *Development of passive immunity against SARS-CoV-2 for management of immunodeficient patients-a perspective.* J Allergy Clin Immunol, 2020. **146**(1): p. 58-60.

8. *Coronavirus drugs trials must get bigger and more collaborative.* Nature, 2020. **581**(7807): p. 120.

9. Bonovas, S. and D. Piovani, *Compassionate Use of Remdesivir in Covid-19.* N Engl J Med, 2020. **382**(25): p. e101.

10. Fätkenheuer, G. and J. Lundgren, *Compassionate Use of Remdesivir in Covid-19.* N Engl J Med, 2020. **382**(25): p. e101.

11. Ferner, R.E. and J.K. Aronson, *Remdesivir in covid-19.* Bmj, 2020. **369**: p. m1610.

12. Hoffmann, C., *Compassionate Use of Remdesivir in Covid-19.* N Engl J Med, 2020. **382**(25): p. e101.

13. Wu, J., B. Wu, and T. Lai, *Compassionate Use of Remdesivir in Covid-19.* N Engl J Med, 2020. **382**(25): p. e101.

14. Wang, Y., et al., *Remdesivir in adults with severe COVID-19: a randomised, double-blind, placebo-controlled, multicentre trial.* Lancet, 2020. **395**(10236): p. 1569-1578.

15. Frost, M.T., et al., *The Adaptive COVID-19 Treatment Trial-1 (ACTT-1) in a real-world population: a comparative observational study.* Crit Care, 2020. **24**(1): p. 677.

16. Bakare, L.S. and J.M. Allen, *COVID-19 Therapeutics: Making Sense of It All.* AACN Adv Crit Care, 2020. **31**(3): p. 239-249.

17. WHO, *Therapeutics and COVID-19: Living Guideline*, W.H. Organization, Editor. 2020. p. 1-38.

18. Corrao, S., G. Natoli, and B. Cacopardo, *A Trial of Lopinavir-Ritonavir in Covid-19.* N Engl J Med, 2020. **382**(21): p. e68.

19. Eljaaly, K. and J.A. Al-Tawfiq, *Crushing lopinavir-ritonavir tablets may decrease the efficacy of therapy in COVID-19 patients.* Travel Med Infect Dis, 2020. **38**: p. 101749.

20. Kunz, K.M., *A Trial of Lopinavir-Ritonavir in Covid-19.* N Engl J Med, 2020. **382**(21): p. e68.

21. Havlichek, D., Jr., *A Trial of Lopinavir-Ritonavir in Covid-19.* N Engl J Med, 2020. **382**(21): p. e68.

22. Carmona-Bayonas, A., P. Jimenez-Fonseca, and E. Castañón, *A Trial of Lopinavir-Ritonavir in Covid-19.* N Engl J Med, 2020. **382**(21): p. e68.

23. Pan, H., et al., *Repurposed Antiviral Drugs for Covid-19 - Interim WHO Solidarity Trial Results.* N Engl J Med, 2021. **384**(6): p. 497-511.

24. Lim, J., et al., *Case of the Index Patient Who Caused Tertiary Transmission of COVID-19 Infection in Korea: the Application of Lopinavir/Ritonavir for the Treatment of COVID-19 Infected Pneumonia Monitored by Quantitative RT-PCR.* J Korean Med Sci, 2020. **35**(6): p. e79.

25. Kim, J.Y., *Letter to the Editor: Case of the Index Patient Who Caused Tertiary Transmission of Coronavirus Disease 2019 in Korea: the Application of Lopinavir/Ritonavir for the Treatment of COVID-19 Pneumonia Monitored by Quantitative RT-PCR.* J Korean Med Sci, 2020. **35**(7): p. e88.

26. Riva, A., et al., *Darunavir does not prevent SARS-CoV-2 infection in HIV patients.* Pharmacol Res, 2020. **157**: p. 104826.

27. NIH. *COVID-19 Treatment Guidelines*. Corticosteroids 2021 October 19, 2021 [cited 2021 November 4]; Available from: <https://www.covid19treatmentguidelines.nih.gov/therapies/immunomodulators/corticosteroids/>.

28. Chen, J., et al., *Clinical progression of patients with COVID-19 in Shanghai, China.* J Infect, 2020. **80**(5): p. e1-e6.

29. Fang, X., et al., *Low-dose corticosteroid therapy does not delay viral clearance in patients with COVID-19.* J Infect, 2020. **81**(1): p. 147-178.

30. Boglione, L., et al., *The proper use of corticosteroids for 2019-nCov pneumonia: Towards promising results?* J Infect, 2021. **82**(1): p. e6-e7.

31. Jung, J., et al., *Re: Low-dose corticosteroid therapy does not delay viral clearance in patients with COVID-19.* J Infect, 2020. **81**(2): p. e79-e81.

32. Rasmussen, S.A., et al., *Coronavirus Disease 2019 (COVID-19) and pregnancy: what obstetricians need to know.* Am J Obstet Gynecol, 2020. **222**(5): p. 415-426.

33. Liauw, J., et al., *Antenatal corticosteroids and COVID-19: balancing benefits and harms.* Am J Obstet Gynecol, 2020. **223**(6): p. 956-957.

34. Rasmussen, S.A. and D.J. Jamieson, *Reply.* Am J Obstet Gynecol, 2020. **223**(6): p. 957-958.

35. Horby, P., et al., *Dexamethasone in Hospitalized Patients with Covid-19.* N Engl J Med, 2021. **384**(8): p. 693-704.

36. Caly, L., et al., *The FDA-approved drug ivermectin inhibits the replication of SARS-CoV-2 in vitro.* Antiviral Res, 2020. **178**: p. 104787.

37. Bray, M., et al., *Ivermectin and COVID-19: A report in Antiviral Research, widespread interest, an FDA warning, two letters to the editor and the authors' responses.* Antiviral Res, 2020. **178**: p. 104805.

38. Chaccour, C., et al., *Ivermectin and COVID-19: Keeping Rigor in Times of Urgency.* Am J Trop Med Hyg, 2020. **102**(6): p. 1156-1157.

39. de Castro, C.G., Jr., L.J. Gregianin, and J.A. Burger, *Continuous high-dose ivermectin appears to be safe in patients with acute myelogenous leukemia and could inform clinical repurposing for COVID-19 infection.* Leuk Lymphoma, 2020. **61**(10): p. 2536-2537.

40. Sharun, K., et al., *Ivermectin, a new candidate therapeutic against SARS-CoV-2/COVID-19.* Ann Clin Microbiol Antimicrob, 2020. **19**(1): p. 23.

41. Van Rensburg, R., et al., *Ivermectin for COVID-19: Promising but not yet conclusive.* S Afr Med J, 2021. **111**(3): p. 13187.

42. Rajter, J.C., et al., *Use of Ivermectin Is Associated With Lower Mortality in Hospitalized Patients With Coronavirus Disease 2019: The Ivermectin in COVID Nineteen Study.* Chest, 2021. **159**(1): p. 85-92.

43. Buonfrate, D. and Z. Bisoffi, *Standard Dose Ivermectin for COVID-19.* Chest, 2021. **159**(5): p. 2111-2112.

44. Keller, K. and J. Sussman, *Chronologic Bias, Confounding by Indication, and COVID-19 Care.* Chest, 2021. **160**(1): p. e86-e87.

45. Ortega-Guillén, E., G. Meneses, and E. Coila, *Remarks About Retrospective Analysis of Ivermectin Effectiveness on Coronavirus Disease 2019 (ICON Study).* Chest, 2021. **159**(5): p. 2110-2111.

46. WHO, *Therapeutics and COVID-19: Living Guideline*, W.H. Organization, Editor. 2021. p. 1-60.

47. Xu, X., et al., *Effective treatment of severe COVID-19 patients with tocilizumab.* Proc Natl Acad Sci U S A, 2020. **117**(20): p. 10970-10975.

48. Yang, C., E. Liu, and M. Liu, *Safety concerns regarding concomitant use of tocilizumab and glucocorticoids in COVID-19 patients.* Proc Natl Acad Sci U S A, 2020. **117**(48): p. 30025-30026.

49. Luo, P., et al., *Tocilizumab treatment in COVID-19: A single center experience.* J Med Virol, 2020. **92**(7): p. 814-818.

50. Xu, X., et al., *Reply to Yang et al.: Tocilizumab treatment in COVID-19 patients needs the assessment of the disease severity and timely intervention.* Proc Natl Acad Sci U S A, 2020. **117**(48): p. 30027-30028.

51. Benucci, M., et al., *COVID-19 pneumonia treated with Sarilumab: A clinical series of eight patients.* J Med Virol, 2020. **92**(11): p. 2368-2370.

52. Buonaguro, F.M., I. Puzanov, and P.A. Ascierto, *Anti-IL6R role in treatment of COVID-19-related ARDS.* J Transl Med, 2020. **18**(1): p. 165.

53. Cellina, M., et al., *Favorable changes of CT findings in a patient with COVID-19 pneumonia after treatment with tocilizumab.* Diagn Interv Imaging, 2020. **101**(5): p. 323-324.

54. Di Giambenedetto, S., et al., *Off-label use of tocilizumab in patients with SARS-CoV-2 infection.* J Med Virol, 2020. **92**(10): p. 1787-1788.

55. Fu, B., X. Xu, and H. Wei, *Why tocilizumab could be an effective treatment for severe COVID-19?* J Transl Med, 2020. **18**(1): p. 164.

56. Hassoun, A., et al., *Utilizing tocilizumab for the treatment of cytokine release syndrome in COVID-19.* J Clin Virol, 2020. **128**: p. 104443.

57. Hormati, A., et al., *Are there any association between COVID-19 severity and immunosuppressive therapy?* Immunol Lett, 2020. **224**: p. 12-13.

58. Mazzitelli, M., et al., *Use of subcutaneous tocilizumab in patients with COVID-19 pneumonia.* J Med Virol, 2021. **93**(1): p. 32-34.

59. Wang, Y., Q. Mao, and X. Zhou, *Does tocilizumab have a magical therapeutic effect on COVID-19 patients without obvious adverse reactions?* Proc Natl Acad Sci U S A, 2020. **117**(49): p. 30896-30897.

60. Xu, X., et al., *Reply to Wang et al.: Tocilizumab treatment should be used in a timely manner, at suitable dose, and in suitable patients.* Proc Natl Acad Sci U S A, 2020. **117**(49): p. 30898-30899.

61. Morrison, A.R., et al., *Acute hypertriglyceridemia in patients with COVID-19 receiving tocilizumab.* J Med Virol, 2020. **92**(10): p. 1791-1792.

62. Sharma, B., *Does use of propofol aggravate the inflammatory markers and cause propofol infusion syndrome in intubated cases of severe COVID-19 infections?* J Med Virol, 2021. **93**(4): p. 1865-1868.

63. Greene, R.A., et al., *Effect of Best Practice Advisories on Sedation Protocol Compliance and Drug-Related Hazardous Condition Mitigation Among Critical Care Patients.* Crit Care Med, 2020. **48**(2): p. 185-191.
