# Supplementary material for: A comment-driven evidence appraisal approach to promoting research findings into practice when only uncertain evidence is available
Source: Health Res Policy Syst. 2023 Mar 27;21:25. doi: 10.1186/s12961-023-00969-9 (PMC10042414; doi:10.1186/s12961-023-00969-9)
Supplement: Supplementary file 2 — Additional file 2. Adherence to reporting guidelines. [file 12961_2023_969_MOESM2_ESM.docx]

Finding What Works in Health Care: Standards for Systematic Reviews. Chapter 5 –

Standards for Reporting Systematic Reviews

Although our manuscript is not strictly a systematic review, we refer to the above

reporting guideline for systematic reviews, such as inclusion and exclusion criteria, in

the process of screening evidence and comments.

Comments in PubMed are usually short papers for supporting or refuting claims, or

discussing methods and findings in original articles (evidence). Evidence-Comment

Networks (ECNs) were constructed by linking COVID-19 related commentaries (letters,

editorials, or brief correspondences) to the original articles that they comment on.

We extracted evidence-comment pairs of 6 drugs for COVID-19 on PubMed database

using the publication-comment linkages function. Recommendations in WHO

guidelines were used as gold standard control to validate the accuracy, coverage, and

efficiency of comments in reshaping clinical knowledge claims.
